# Hepatic alterations are accompanied by changes to bile acid transporter-expressing neurons in the hypothalamus after traumatic brain injury

**DOI:** 10.1038/srep40112

**Published:** 2017-01-20

**Authors:** Damir Nizamutdinov, Sharon DeMorrow, Matthew McMillin, Jessica Kain, Sanjib Mukherjee, Suzanne Zeitouni, Gabriel Frampton, Paul Clint S. Bricker, Jacob Hurst, Lee A. Shapiro

**Affiliations:** 1 Department of Surgery, Texas A&M University Health Science Center, College of Medicine, Temple, Texas, 76504, USA; 2 Department of Neurosurgery, Neuroscience Research Institute, Baylor Scott & White Health, Temple, Texas, 76504, USA; 3 Departent of Internal Medicine, Texas A&M University Health Science Center, College of Medicine, Temple, Texas, 76504, USA; 4 Central Texas Veterans Health Care System, Temple, Texas, 76504, USA.

## Abstract

Annually, there are over 2 million incidents of traumatic brain injury (TBI) and treatment options are non-existent. While many TBI studies have focused on the brain, peripheral contributions involving the digestive and immune systems are emerging as factors involved in the various symptomology associated with TBI. We hypothesized that TBI would alter hepatic function, including bile acid system machinery in the liver and brain. The results show activation of the hepatic acute phase response by 2 hours after TBI, hepatic inflammation by 6 hours after TBI and a decrease in hepatic transcription factors, Gli 1, Gli 2, Gli 3 at 2 and 24 hrs after TBI. Bile acid receptors and transporters were decreased as early as 2 hrs after TBI until at least 24 hrs after TBI. Quantification of bile acid transporter, ASBT-expressing neurons in the hypothalamus, revealed a significant decrease following TBI. These results are the first to show such changes following a TBI, and are compatible with previous studies of the bile acid system in stroke models. The data support the emerging idea of a systemic influence to neurological disorders and point to the need for future studies to better define specific mechanisms of action.

Traumatic brain injury (TBI) represents a serious socioeconomic concern. There are over 2 million reported cases of TBI each year and treatment options are lacking. Due to the nature of its direct impact in the brain, diagnosis and treatment of TBI has been targeted towards neurological mechanisms. There is an extensive literature regarding the numerous anatomical and molecular changes that are observed in the central nervous system (CNS) following head trauma. However, there is also evidence that systemic mechanisms engaged after TBI may negatively impact neurological function^1^.

Neuroinflammation, believed to be a key player in the neuropathological response to TBI, may also influence systemic alterations^2^,^3^,^4^,^5^,^6^. Following a TBI, a number of chemokines and cytokines are released in the brain as part of the neuroinflammatory response^7^,^8^. Interestingly, neuroinflammatory responses have previously been associated with significant changes to the expression of almost a thousand genes in the liver^9^. Moreover, some of the cytokines typically seen elevated in the brain across a wide range of TBIs include, tumor necrosis factor-α (TNF-α), interleukin-1β (IL-1β), interleukin-6 (IL-6), and interferon (IFN)^10^,^11^,^12^,^13^. Three of these aforementioned cytokines (TNF-α, IL-1β. IL-6) are known as acute phase response effector proteins. Previous studies have demonstrated that these acute phase effector proteins are elevated in the brain and in serum after TBI, and that elevated serum levels result in binding to hepatocytes, thus stimulating the hepatic acute-phase response (APR)^9^,^14^.

The APR is a major component of the systemic response to tissue damage, infection, inflammation and trauma, including neurotrauma^15^,^16^. The APR is coordinated primarily by the liver^17^ via the release of regulatory acute-phase proteins^9^. The positive acute-phase proteins include, C-reactive protein (CRP), haptoglobin, serum amyloid A (SAA), and serum amyloid P (SAP)^18^,^19^,^20^. Once released, these proteins modulate the inflammatory response, stimulate the complement system, scavenge free radicals and neutralize enzymatic activity^16^. The collective action of initiation of the APR is intended to minimize tissue damage and initiate repair^21^,^22^,^23^. Considering that we and others have demonstrated elevated levels in the brain of the acute phase effector proteins, TNF-α, IL-1β and IL-6 after a TBI^7^,^24^, and the fact that elevated CNS cytokines are rapidly followed by elevated levels in the systemic circulation^21^,^22^,^23^, we hypothesized that there would be elevated acute phase response proteins in the liver, indicative of a hepatic APR after TBI.

To further assess hepatic alterations after TBI, we examined hedgehog signaling. Increased hedgehog signaling in the liver has been found during many types of liver injury^25^. Hedgehog signaling is comprised of several hedgehog molecules including Sonic hedgehog (Shh) and Indian hedgehog (Ihh). Hedgehog signaling results in the nuclear localization of the Gli family (Gli 1, Gli 2, Gli 3) of transcription factors^26^,^27^,^28^. The cumulative effect of activating hedgehog pathways includes the accumulation, mobilization and activation of inflammatory cells^25^, including B^29^ and T cells^30^, as well as regulating the expression of Gli target genes^31^. Thus, this study assessed gene expression of hedgehog and Gli in the liver, in order to provide insight into whether or not TBI alters hepatic functioning. Hepatic inflammation was also assessed at the level of transcription and translation.

The bile acid system may represent another hepatic system that is altered following a TBI^32^. In humans and mice, bile acids are predominantly synthesized in the liver and stored in the gall bladder (rats lack a gall bladder, but have an intestinal modification to serve similar functions). The molecular machinery for bile acid synthesis and metabolism are present in the CNS, as are some of the bile acid transporters and receptors^33^,^34^,^35^,^36^. Bile acid transporters are central to maintaining bile acid circulation and homeostasis^37^, and specific transporters carefully regulate entry and removal from the CNS. Blood brain barrier (BBB) breakdown can facilitate bile acid entry into the brain^38^. Once in the brain, bile acids can be beneficial^39^ or detrimental^32^,^38^ depending on the physiological conditions, as well as the type and location of the receptors to which they bind^40^. Bile acids receptors may be cell-surface or nuclear, and have tissue-specific effects related to cholesterol synthesis, bile acid synthesis, and other g-protein mediated effects^41^,^42^.

Alterations to the hepatic and/or brain bile acid systems have been shown to influence neurological function in several different animal models. For example, in an acute liver failure model, bile acid signaling was shown to be involved in neurological decline^32^,^43^,^44^. Other studies using stroke models have demonstrated that the bile acid, tauroursodeoxycholic acid, reduces apoptosis and is neuroprotective after stroke^45^,^46^. Moreover, McMillin M *et al*.^40^, have shown in a model of cholestasis, that bile acid signaling was capable of suppressing the hypothalamic-pituitary axis (HPA). It is pertinent to note that the HPA is known to be dysfunctional following TBI^47^,^48^.

Considering the importance of the bile acid receptors and transporters in mediating bile acid signaling, as well as the fact that previous studies illustrate direct bile-acid mediated neurological effects, the current study was designed to test the hypothesis that TBI results in alterations to the bile acid receptors and transporters in the liver and hypothalamus. Our working model for this hypothesis is shown in Fig. 1. Briefly, we pose that the increase in IL-1β, IL-6 and TNF-α after TBI results in activation of the hepatic APR. The APR precedes hepatic inflammation and altered bile acid release. Because a TBI results in blood brain barrier breakdown, bile acids enter the brain in concentrations outside of the typical regulation by the bile acid transporters. Thus, dysregulation of brain bile acid homeostasis after TBI may contribute to detrimental neurological outcomes.

## Results

### Lateral fluid percussion injury (FPI) selectively elevates hepatic acute phase proteins

**Figure 2:**
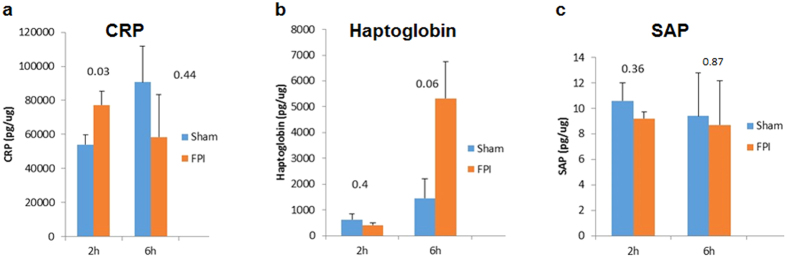
Multiplex assay of acute phase response protein levels in the liver. In (**a**) C Response Protein (CRP) is significantly elevated in the liver at 2 hrs following FPI (P < 0.04) FPI, but not at 6 hrs after FPI in mice. In (**b**), analysis of haptoglobin levels in the liver after FPI revealed no significant difference at 2 hrs after FPI, and a trend towards significance (P = 0.061) at 6 hrs after FPI. In (**c**), analysis of serum amyloid P component (SAP) revealed no significant differences at 2 or 4 hrs after FPI. Taken together, these results demonstrate differential elevations of acute phase proteins in the liver following an FPI. Such data highlights the importance of examining multiple acute phase proteins, at several time points in order to detect activation of the APR. Data are presented as means ± the standard error of the mean (SEM). Experiments were performed in triplicate at minimum, and a value of P =< 0.05 was considered statistically significant.

Because we have previously demonstrated elevation in the brain of acute phase effector proteins^7^, we assessed whether there would also be an acute phase response in the liver. Therefore, we assayed acute phase proteins in the liver at 2 and 6 hrs after FPI. The results showed that CRP is significantly elevated in the liver at 2 hrs following TBI (F(1,14) = 5.56, P < 0.04), but not at 6 hrs after TBI (Fig. 2a). Analysis of haptoglobin showed a trend toward significant elevation in the liver at 6 hrs after TBI (F(1,14) = 4.033, P = 0.061, NS), however there were no significant differences at 2 hrs after TBI (Fig. 2b). Analysis of serum amyloid protein revealed no significant differences at either of the examined time points (Fig. 2c).

### Hepatic inflammation after FPI

**Figure 3:**
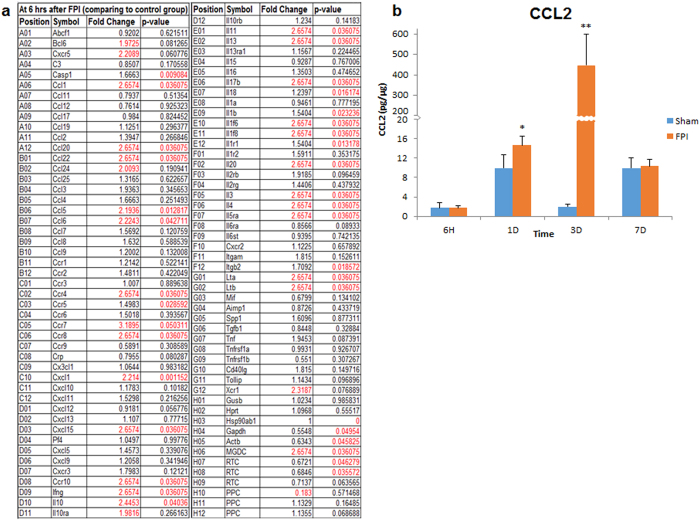
Hepatic inflammation after FPI. In (**a**), results from an inflammatory PCR array panel run from liver homogenates at 6 hrs after FPI is shown. Numbers in red font color depict significantly different (P < 0.05) from sham. In (**b**), Inflammatory protein levels of CCL2 are shown at 6 hrs, and 1, 3 and 7 days after FPI. The results show that CCL2 in the liver is significantly increased at 1 (P < 0.05) and at 3 (P < 0.003) days after TBI, but not at 6 hrs or 7 days after FPI. CCL2 is commonly used as a general indicator of inflammation and the peak increase at 3 days post-FPI mirrors our previous result in the brain^25^. Data are presented as means ± SEM. Experiments were performed in triplicate at minimum, and a value of P =< 0.05 was considered statistically significant. *< 0.05; **< 0.005.

Because the APR is associated with hepatic inflammation, we next examined inflammatory cytokines and chemokines in the liver by polymerase chain reaction (PCR). We hypothesized that the message for inflammatory protein translation in the liver would be initiated very early after TBI, because it rapidly follows the APR. The results from the analysis of a cytokine/chemokine PCR array are shown in Fig. 3. Numerous significant changes were detected using the inflammatory panel at 6 hrs after TBI (Fig. 3a). In order to determine if the increased ribonucleic acid (RNA) was subsequently accompanied by increased expression of inflammatory proteins, we performed a multiplex analysis of several cytokines in the liver in different time points after TBI. The results revealed significant elevation of chemokine (CC motif) ligand 2 (CCL2) in the liver beginning at 1 day after FPI (t(5) = 4.864; P < 0.05), reaching a maximum level at 3 days after FPI (t(5) = 10.040; P < 0.0001). No significant difference was observed at 6 hours or 7 days after FPI (Fig. 3b). We also found no significant differences in custom cytokine panel consisting of CCL2, chemokine (CC motif) ligand 5 (CCL5), chemokine (C-X-C motif) ligand 1 (CXCL1), or interleukin-4 (IL-4), although a trend toward increased CCL5 and IL-4 were observed at 6 hrs after FPI, compared to shams (Not shown).

### Hedgehog and Gli alterations in the liver after FPI

**Figure 4:**
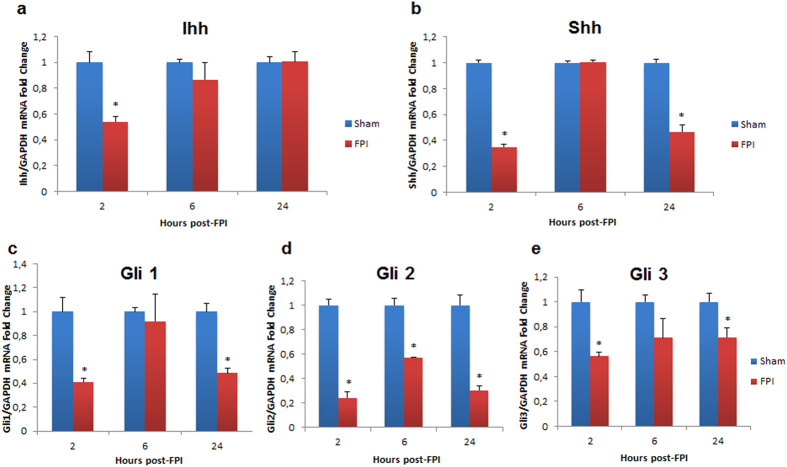
Expression levels of hedgehog and Gli proteins in the liver at 2, 6 and 24 hours after FPI in mice. In In (**a**), Indian hedgehog (Ihh) is significantly decreased at 2 hrs after FPI in mice, but not at 6 or 24 hrs post-FPI. In (**b**), sonic hedgehog (Shh) expression is decreased at 2 and 24 hrs after TBI, but not at 6 hrs. In (**c**–**e**), Gli 1, 2 and 3 are all significantly decreased at 2 and 24 hrs after FPI. Gli 2 is also significantly decreased at 6 hrs after FPI, whereas no differences are found at this time point for Gli 1 and Gli 3. Data are presented as means ± SEM. Experiments were performed in triplicate at minimum, and a value of P =< 0.05 was considered statistically significant. *< 0.05.

Because hedgehog signaling is associated with accumulation and mobilization of immune cells in the liver, and because Gli expression is an indicator of hedgehog activation, we examined levels of messenger ribonucleic acid (mRNA) expression of hedgehog homologues and their effectors, the Gli family proteins, in the liver at 2, 6, and 24 hrs after FPI. Ihh mRNA levels significantly decreased (vs. sham, P < 0.05) at 2 hrs after TBI, but not at 6 or 24 hrs post-TBI (Fig. 4a). Shh expression levels were significantly decreased at 2 and 24 hrs after (vs. sham, P < 0.05), but not at 6 hrs (Fig. 4b). Gli 1, Gli 2 and Gli 3 were all significantly decreased at 2 and 24 hrs time points after FPI (Fig. 4c–e). Gli 2 was also significantly decreased at 6 hrs after FPI, whereas no significant differences were found at this time point for Gli 1 and Gli 3 (Fig. 4c–e).

### Hepatic bile acid system changes after FPI

**Figure 5:**
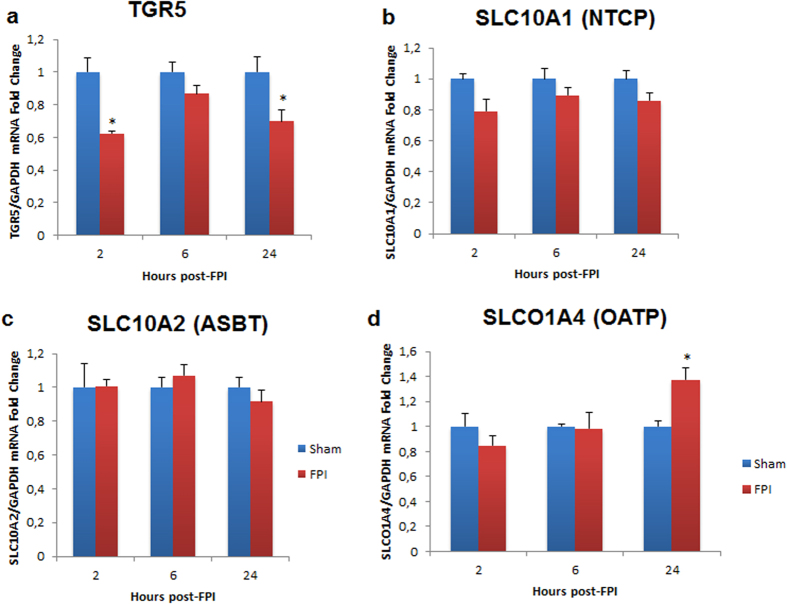
Bile acid receptor and transporter expression in the liver at 2, 6 and 24 hours after FPI. In (**a**), TGR5 is significantly decreased at all three time points after FPI. In (**b**,**c**), no changes are seen for NTCP or ASBT. In (**d**), OATP is significantly elevated at 24 hrs after FPI, but not at 2 or 6 hrs after FPI. Taken together, the data show altered bile acid system in the liver after FPI. Data are presented as means ± SEM. Experiments were performed in triplicate at minimum, and a value of P =< 0.05 was considered statistically significant. *P < 0.05.

Considering that alterations to hepatic function have been associated with changes to the bile acid system, we next examined bile acid receptors and transporters in the liver following FPI. The results showed that G-protein coupled bile acid receptor-1 (TGR5) was significantly decreased at 2 and 24 hrs after TBI and a trend towards reduced TGR5 was observed at 6 hrs after TBI (Fig. 5a). Alternatively, no significant differences were observed for apical sodium dependent bile acid transporter (ASBT) or sodium/bile acid cotransporter (NTCP) at any of the time points examined (Fig. 5b,c). Expression level of solute carrier organic anion transporter family member 1A4 (OATP) was significantly increased (vs. sham, P < 0.05) at 24 hrs time point after TBI, without significant changes at 2 and 6 hrs (Fig. 5d).

### Brain bile acid system changes after FPI

**Figure 6:**
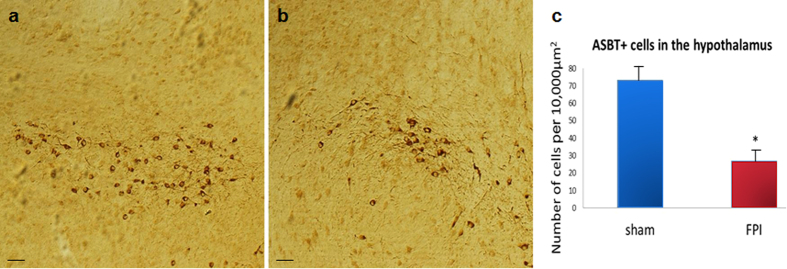
ASBT immunohistochemistry in the hypothalamus at 7 days after FPI in mice. We developed a custom antibody that allows for the identification of neuronal expressed ASBT in the brain. In (**a**), ASBT staining in the dorsal medial nucleus of the hypothalamus of a sham mouse is shown 7 days after sham FPI. In (**b**), a significant decrease in ASBT-labeled cells is observed in the dorsal medial nucleus of the hypothalamus at 7 days FPI. In (**c**), stereological analysis of ASBT+ cells in the hypothalamus of sham and FPI (mild/moderate) mice shows a significant (P < 0.05) decrease at 7 days after FPI. These results support the hypothesis that TBI induces alterations to the bile acid system in the liver (Fig. 5) and in the brain. Data are presented as means ± SEM. P =< 0.05 was considered statistically significant. Scale bars in A and B = 50 μm.

Recent evidence also demonstrates the presence of bile acid machinery in the CNS^40^,^49^. We performed immunohistochemistry for the bile acid transporter ASBT and found that ASBT-expressing neurons were predominately located in the hypothalamus (Fig. 6a,b), with a smaller number of ASBT-expressing neurons located in the raphe nucleus. We performed quantitative analysis of the ASBT-expressing neurons on day 7 in the hypothalamus of sham (Fig. 6a) and after TBI (Fig. 6b). We observed ASBT+ neurons in the medial and lateral preoptic area, the dorsal medial nucleus of the hypothalamus and the lateral hypothalamus. Although none of these regions showed significant changes when analyzed separately (not shown), when we pooled the three regions, we observed a significant loss of ASBT-expressing neurons following TBI (t(9) = 6.64, P < 0.05), compared to shams (Fig. 6c). We also observed ASBT-expressing neurons in zone incerta, subparafacicular thalamic nucleus and periaqueductal gray area. We did not detect any significant changes in these regions after TBI (not shown).

## Discussion

The results from this study demonstrate several hepatic, inflammatory, and bile acid system changes, including changes in the brain bile acid machinery, following FPI. These changes include elevation of hepatic acute phase proteins, hepatic inflammation, alterations to bile acid receptors and transporters in the liver, and a loss of ASBT+ neurons in the hypothalamus. Previous studies using various brain injury models have demonstrated some aspects of hepatic alterations^50^,^51^,^52^. However, this study is unique in that it is more comprehensive, and is the first to demonstrate alterations to the bile acid system in the liver and brain following TBI.

The APR is typically initiated in response to most inflammatory triggers in order to coordinate the innate immune response^53^,^54^,^55^. A major role for the liver in initiating the APR and mediating the inflammatory response has been well-described^17^,^18^,^19^,^20^. Triggers for the APR include elevations of the cytokines IL-1β, IL-6 and TNF-α, which can bind directly to hepatocytes and induce the synthesis and subsequent release of acute phase response proteins. Previous studies are inconsistent in regards to TBI activating the APR; where some studies support TBI activation of the APR^20^,^52^,^56^,^57^, and others do not^58^. It is possible that some of these discrepancies can be explained by differential sensitivity of the markers, lack of temporal specificity, not examining enough of the proteins, or that not all brain injuries induce the APR. Nevertheless, the current study provides additional evidence for activation of the APR after TBI, as well as data that are consistent with previous reports of hepatic inflammation following various types of brain injury^50^,^51^,^52^.

In addition to modulating the APR, the liver also controls the production of bile acids. Bile acids have been reported throughout the brain^32^,^40^ and bile acid receptor and transporter expression in the brain has also been established^32^,^39^,^42^,^59^,^60^,^61^. The fact that bile acid receptors and transporters are found throughout the brain suggests the possibility that bile acids might be able to modulate neuronal function^38^. Of particular interest to the current study, bile acid receptors and transporters are found in the hypothalamus and hypothalamic function seems to be particularly sensitive to bile acid modulation^36^,^40^,^62^. Support for a role of bile acid receptor activation on neurological function was demonstrated by data showing that the bile acid chenodeoxycholate (CDCA) is a potent antagonist of N-Methyl-D-aspartic acid (NMDA) and gamma-aminobutyric acid^63^. CDCA significantly decreased the firing rate of hypothalamic neurons and synchronized network activity^63^.

Within the hypothalamus, we found ASBT+ neurons in medial and lateral portions of the preoptic area, the dorsal medial nucleus and the lateral hypothalamus. The dorsal medial nucleus is involved in regulating circadian rhythm, feeding, and drinking behavior, as well as body metabolism^64^,^65^. Considering that sleep cycles are often altered following TBI, it is possible that dysregulation of the bile acid system in this nuclei is involved in this alteration. Interestingly, the lateral portions of the preoptic nuclei, and the lateral hypothalamus, have also been shown to be involved in sleep onset^66^,^67^. Considering that we observed ASBT+ neurons in these hypothalamic nuclei, it is possible that bile acid system changes after TBI might be involved in sleep and/or circadian deficits after TBI.

Further evidence supporting a mediating role of the bile acid system on neurological function is obtained from studies showing that the bile acids tauroursodeoxycholic acid (TUDCA) and ursodeoxycholic acid (UDCA) are neuroprotective in protein misfolding disease models, including Parkinson’s, Huntington’s and Alzheimer’s diseases^49^. It is also now clear that bile acids function as nutrient hormones that can activate specific nuclear receptors, G-protein coupled receptors^68^, and cell signaling pathways in a physiological relevant manner^69^. Therefore, the bile acid system can mediate neurological function through multiple mechanisms, and the homeostasis of this system is altered following TBI.

The finding of decreased hedgehog and Gli mRNA in the liver early after FPI is somewhat surprising, and as mentioned in the methods, may be underestimated. A decrease in these signaling molecules in the liver results in a decrease in proliferation of hepatocytes. Alternatively, an increase in hedgehog and Gli signaling is associated with inflammation and hepatic repair. What is interesting is the apparent cyclical pattern of the results, suggesting a possible biphasic response in the liver at early time points after TBI. Previous *in vitro* and *in vivo* studies examining acute liver injury have demonstrated a biphasic hepatocyte response that may correspond to hepatocyte apoptosis and/or liver necrosis^70^,^71^. Thus, it is possible that at later, or different time points than we examined, hedgehog and Gli signaling are increased, as the liver mobilizes immune cells and attempts to restore homeostatic function^72^. Future studies are needed to clarify this disparity.

In conclusion, this study provides ample and novel evidence supporting TBI-induced alterations to the liver and to the bile acid system, through several systems. Considering the findings that the bile acid system can be manipulated to alter neuronal function, it is possible that targeting the bile acid system could be useful in treating TBI. Future studies are needed to explore the potential interactions between the liver and brain, and to determine if manipulating liver/brain interactions after TBI will improve outcomes.

## Methods

### Fluid percussion traumatic brain injury

Procedures on research animals were conducted with approval of the Baylor Scott & White Animal Care and Use Committee in accordance with the recommendations of the Guide for the Care and Use of Laboratory Animals (Eighth edition) revised by the National Research Council in 2011 (published by the National Academies Press), National Institutes of Health and American Association for the Accreditation of Laboratory Animal Care (AAALAC) guidelines and the Public Health Service (PHS) Policy on Humane Care and Use of Laboratory Animals.

A FPI was induced (~1.2 atm) in male C57/BL6 mice (~22–26 g) as previously described^73^. Sham mice received identical treatment as the FPI mice that included isoflurane anesthesia and craniotomy. As craniotomy alone induces low levels of inflammation, we use sham mice that receive the identical craniotomy procedure, but do not receive the FPI. All mice were group housed prior to procedures. Thereafter, mice were individually housed in a 12:12 light dark cycle, with food and water available ad libitum. We had no mortality and all mice were included in the study. At 2 hrs (n = 3 sham & n = 3 FPI), 6 hrs (n = 11 sham & n = 11 FPI), 24 hrs (n = 12 sham & n = 12 FPI), 3 days (n = 8 sham & n = 8 FPI), and 7 days (n = 6 sham & n = 6 FPI) after injury, mice were anesthetized with isoflurane, livers were rapidly dissected, and flash-frozen. In a separate group of mice used for immunohistochemistry (N = 5 sham and n = 5 FPI), mice were perfused at 7 days post-FPI, as previously described^73^.

### Real-time PCR

RNA was extracted from liver tissue (n = 3 sham & 3 FPI per time point) and real-time PCR was performed as previously described^74^ using commercially available primers against mouse ASBT, TGR5, OATP, NTCP, Shh, Ihh, Gli 1, Gli 2, Gli 3 and glyceraldehyde 3-phosphate dehydrogenase (GAPDH) (Qiagen, Frederick, MD). For these experiments, we used GAPDH as our loading control. It is pertinent to note that a two-tailed t test revealed a trend toward decreased GAPDH in the FPI group, compared to sham. Therefore, it is possible that the magnitude of change in hedgehog and Gli are underestimated, although the interpretations remain unchanged. The primers were purchased from commercially available source (SABiosciences; Cat. Number: PPR06557B). The RT-PCR inflammatory array has multiple housekeeping genes and in this experiment heat shock protein 90 kDa Alpha Family Class B Member 1 (HSP90AB1) was found to be the most stable. Thus, the loading control used for analysis of this panel was HSP90AB1. In addition, we used an inflammatory cytokines and receptors RT^2^ profiler PCR Array system (Qiagen, Frederick, MD) following manufacturer’s protocols including analysis using their supplied software. For statistical analysis, the p values were calculated as previously described^75^ based on a Student’s t-test of the replicate 2^ (−Delta CT) values for each gene in the sham-treated groups and treatment groups with p values less than 0.05 considered significant. Data are expressed as mean relative mRNA levels +/−SEM.

### Multiplex Assays for acute phase proteins and cytokines

Frozen liver tissue (n − 8 sham & 8 FPI at 6, 24 and 72 hrs post-FPI; n = 6 sham & n = 6 FPI at 7 days post-FPI) was homogenized in purification detergent buffers of ProteoExtract® Cytoskeleton Enrichment and Isolation Kit (EMD Millipore, Billerica, MA) using MACS tissue homogenizer with protein setting. Then the sample is spun down at 1,000 G for 5 min and stored at −20 °C. Multiplex assays were done as previously described in a previous study^7^ using the manufacturer’s protocol for both the cytokines (Millipore) acute phase protein analysis (Millipore). We used a custom acute phase panel containing CRP, haptoglobin and SAP (*currently available as part of kit: MAP2MAG-76K). For multiplexing, we used a custom cytokine panel consisting of CCL2, CCL5, CXCL1, IL-3 and IL-4. It should be noted that the IL-3 measurements were below the range of detection for the multiplex and no statistics were performed for this cytokine. For both sets of analysis, all assays were performed in triplicate and the averages of the 3 were used for statistical analysis. Statistical analysis was performed using one way ANOVA to compare sham vs. FPI at each time point.

### Stereological analysis of immunocytochemical staining of ASBT-labeled hypothalamic neurons

Mice (n = 5 sham & n = 5 FPI) were perfused at 7 days after FPI, with saline followed by 4% paraformaldehyde and post-fixed, as previously described^7^. Serial sections were cut at 50 microns, using a vibratome sectioning system, as previously descried^7^. We selected every 6^th^ section for stereological analysis of the hypothalamus. This brain region was selected because altered serum bile acids have been shown to influence the hypothalamic-pituitary axis^40^ and because the ASBT antibody selectively stains a population of hypothalamic neurons. ASBT antibody was generated (Pacific Immunology; Ramona, CA) as previously described^76^. Tissue was first rinsed 3 times for 5 min each in phosphate-buffered saline (PBS), after which it was soaked in 0.5% solution of hydrogen peroxide (H_2_O_2_) in PBS for 30 min, then for 60 min in 1.0% H_2_O_2_ in PBS, followed by 30 min in 0.5%v H_2_O_2_ in PBS. After rinsing 3 times for 5 min each in PBS, the tissue was incubated free-floating, rotating for 24 hrs, in anti-ASBT (1:200), with 0.001% triton-X, and normal goat serum (1:20) in PBS, at room temperature (RT). Tissue was then rinsed in PBS 3 times for 5 min each, after which is was incubated for 60 min by rotating at RT in biotinylated anti-rabbit IgG (1:200; Vector Labs, Burlingame, CA) in PBS with normal goat serum (1:20) and 0.001% triton-X. Tissue was rinsed in PBS 3 times for 5 min each, and then incubated in ABC kit as per manufacturer instructions (Vector Labs, Burlingame, CA) for 1 hr, rotating at RT. Tissue was rinsed in PBS and then reacted in the DAB kit, as per manufacturer instructions (Vector Labs, Burlingame, CA). A Student’s T test was used to compare sham and FPI groups.

### Statistical analysis

Data are presented as means ± the standard error of the mean (SEM). Significant differences among groups were estimated as described and a value of P < 0.05 was considered statistically significant.

## Additional Information

**How to cite this article**: Nizamutdinov, D. *et al*. Hepatic alterations are accompanied by changes to bile acid transporter-expressing neurons in the hypothalamus after traumatic brain injury. *Sci. Rep.* **7**, 40112; doi: 10.1038/srep40112 (2017).

**Publisher's note:** Springer Nature remains neutral with regard to jurisdictional claims in published maps and institutional affiliations.

## Figures and Tables

**Figure 1:**
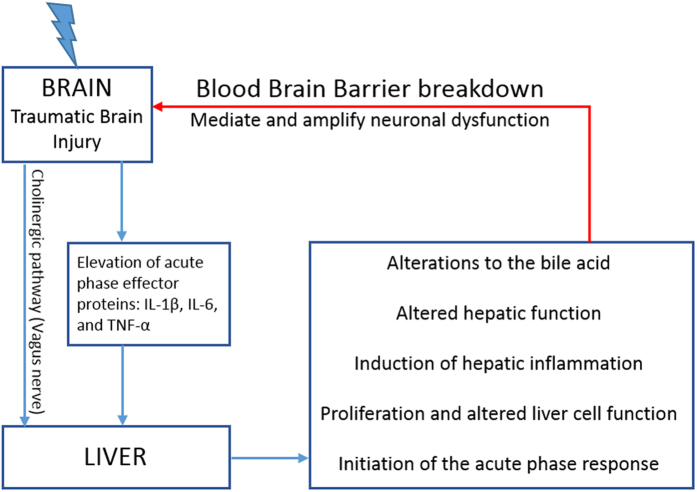
Proposed mechanisms of hepatic influences to TBI. An injury to the brain stimulates a hepatic response via (A) cholinergic inputs and (B) via increased serum concentrations of the acute phase effector proteins, IL-1β, IL-6 and TNF-α. Serum elevations of these proteins bind to hepatocytes in the liver and stimulate production in acute phase proteins. This increased production of acute phase proteins is associated with altered liver cell function, including changes in protein synthesis, proliferation and metabolic function. As part of the acute phase response, leukocytosis occurs that is associated with an increase in the production of inflammatory cytokines and chemokines by the liver. Functionally, the collective alterations to liver cell function and hepatic inflammation result in changes to the bile acid milieu, bile acid receptors and bile acid trasnsporters. We hypothesize that because of blood-brain-barrier breakdown, any one or more of these hepatic mechanisms can influence neuronal dysfunction following TBI.
